# Therapeutic management of severe hypothermia with veno-arterial ECMO: where do we stand? Case report and review of the current literature

**DOI:** 10.1186/s13049-020-00723-y

**Published:** 2020-04-21

**Authors:** Aurélien Ledoux, Piehr Saint Leger

**Affiliations:** Department of Intensive Care Medicine, General Hospital of Valenciennes, Valenciennes, France

**Keywords:** Hypothermia, Cardiac arrest, Extracorporeal membrane oxygenation, Rewarming

## Abstract

**Background:**

Severe accidental hypothermia is associated with high morbidity and mortality. Veno-arterial extracorporeal membrane oxygenation (VA-ECMO) provides an efficient rewarming method with complete cardiopulmonary support. The use of VA-ECMO for this indication has greatly improved the vital and functional prognosis of patients.

**Case presentation:**

We report a case of a 46-year-old patient who was treated for severe hypothermia with a temperature of 22.4 °C along with initial cardiac arrest, whose progression was favorable after the implementation of VA-ECMO support. Two months after initial cardiac arrest, the patient was reassessed and showed signs of complete recovery with regard to his mental and physical capacities.

**Conclusions:**

The recent international publications and groups of experts recommend the use of VA ECMO as the gold standard therapy to treat severe hypothermia. Therefore, it seems suitable to update the current knowledge on the topic by analysing the latest international publications. The performance of this technique calls into question ethical and economic factors. Two distinct medical teams tried to identify and regroup prognosis factors in predictive survival scores. They raise the question of the utility of these scores in clinical practice. Indeed, according to which survival rate should we proceed to prolonged resuscitation and implement VA-ECMO? Additional studies will be needed for external approval of these survival scores, and additional reflection by experts will be required.

## Introduction

Severe accidental hypothermia is defined as a core body temperature below or equal to 28 °C corresponding to hypothermia degree III (HT III) and IV (HT IV) in the Swiss staging model of the international classification of accidental hypothermia, presented in Table 1 [1]. In these hypothermia, HT III and HT IV, severe hemodynamic instability can lead to cardiac arrest, with increased mortality from deeper hypothermia, but no percentage of mortality exists in the literature.

**Table 1:** Swiss clinical staging of hypothermia [1]

Stage	Clinical symptoms	Typical core temperature (°C)
Hypothermia I	Conscious, shivering	35–32 °C
Hypothermia II	Impaired consciousness, no shivering	32–28 °C
Hypothermia III	Unconscious, no shivering, vitals signs present	28–24 °C
Hypothermia IV	No or minimal vital signs	< 24 °C

The treatment in these indications are based on active internal warming by extracorporeal life support (ECLS) with the use of vein-arterial ECMO (VA-ECMO) which is increasingly recommended as the preferred method due to its rapid availability, lower heparinization requirement and possibility of prolonged support [2, 3].

First, we will describe a case study of a positive outcome of a patient who was treated for HT IV with early VA ECMO. Then, we will explore and review the available literature, discuss it and, finally, propose some recommendations.

## Report

A 46-year-old male was found in the street during winter in a calm areactive coma. The man was still breathing, but his core body temperature was 22.4 °C. The patient presented cardiovascular instability due to ventricular fibrillation during secure mobilization and was eligible for cardiopulmonary resuscitation (CPR). Return of spontaneous circulation (ROSC) occured after 45 min of cardiac massage, 7 external electric shocks and 10 mg of intravascular epinephrine. Bradycardia remained at 30 beats per minute, leading to severe hypotension, bilateral mydriasis and Glasgow score of 3. He received no sedative medicine.

The initial balance sheet showed severe acidosis with a pH level of 6.98 combined with hyperlactatemia of 14.2 mmol/L. The serum potassium level was 4 mmol/L. Disseminated intravascular coagulation was observed with a prothrombin ratio of 54%, fibrin monomers at 122 μ g/ml and D-Dimers at 15,1 mg/L. Liver function tests were abnormal, with AST 25-times higher than normal. Rhabdomyolysis was observed at CPK 6.700 U/L. Troponin at 0,029 μg/l was in the normal range (*N* < 0,04 μg/L). Kidney function was disturbed, evidenced by a creatinine concentration of 244 μmol/L.

The decision to apply extracorporeal life support (ECLS) was based on the following criteria: severity of core hypothermia, range of serum potassium level and occurrence of witnessed cardiac arrest with immediate successful CPR. Femoro-femoral VA-ECMO was performed in the ward upon arrival in order to provide active rewarming and circulatory support. The time from initial cardiopulmonary arrest to functional VA-ECMO was approximately 260 min. The sweep gas flow and ECMOFiO_2_ were adjusted based on arterial blood gas analysis.

Return to a core body temperature above 36 °C occured within 40 min of VA-ECMO implementation. There were no cardiac-related complications under VA-ECMO. Cardiac function was assessed by echocardiography, and no systolic dysfunction was reported on day 2. Left ventricular ejection fraction (LVEF) was 55%, and sub-aortic velocity time integral (VTI) was 19 cm/s. VA-ECMO withdrawal was reviewed and performed on day 2.

The patient stopped receiving sedatives on day 4 in order to perform neurologic evaluation. The patient presented generalized myoclonic seizures. Symptomatic treatment was achieved by levetiracetam after magnetic resonance imaging of the head revealed a minor post-anoxic injury. During his recovery, the patient acquired ventilator-associated pneumonia, documented by *Pseudomonas aeruginosa* infection, which delayed extubation to day 17. There was progressive recovery of renal function without the requirement of renal replacement therapy.

On day 26, the patient was transferred to the respiratory rehabilitation treatment. The patient was discharged from the hospital on day 40.

The patient was reassessed 2 months after the initial report. Clinical examination revealed a fully conscious and oriented patient, with disappearance of myoclonic seizures. He was able to walk without any help. Functional respiratory tests did not reveal any sequelae. Control echocardiography was performed, and the results showed a LVEF of 60% without any diastolic dysfunction.

## Discussion and literature review

Severe hypothermia has multiple pathophysiological consequences within the organism, mostly affecting the cardiovascular system (heart conduction system, sinus bradycardia, long QT, J Osborne wave, ventricular fibrillation, severe hypotension due to vasoplegia and decreased cardiac output) [4–7], but also other systems such as the respiratory, cerebral or metabolic systems. All of these disorders lead to death of the patient as a result of cardiac or respiratory arrest, unless adequate treatment is provided.

HT III and HT IV treatment is based on warming the patient in order to restore homeostasis of the different systems and internal organs. In these cases, ECLS, which replaces the function of the heart by a pump and the function of the lungs by an oxygenation membrane, with a control of the temperature of the circuit, is the only recommended device to provide active internal warming [1, 2]. Without any active internal warming, active external warming techniques are not used due to the risks associated with low cardiac output caused by peripheral vasodilatation. When ECLS is not available, other active internal warming techniques should be considered, such as peritoneal lavage, intravascular warming catheter and renal replacement therapy (intermittent or continuous) [2, 3].

Among the available ECLS devices, central or peripheral cardiopulmonary bypass (CPB) was the first replacement technique used in the early 1980s, with several conclusive publications on the topic. In particular, Althaus et al. reported full recovery of 3 patients after hypothermic cardio-respiratory arrest. One of the patients underwent active central warming by a central shunt, whereas the other two patients had a peripheral shunt with a femoro-femoral approach [8]. Since the early 2000s VA-ECMO has been the gold standard therapy for this indication [6, 7]. Compared to central cardio-pulmonary shunt, VA-ECMO remains a faster and easier supportive treatment to implement. Moreover, it requires less anticoagulation to function and has the ability to be maintained in place for effective support in the event of persistent cardiac instability or severe pulmonary oedema after rewarming. Ruttmann et al. demonstrated this use in a retrospective series of 59 patients with severe hypothermia with cardio-circulatory arrest at admission. Despite a high mortality rate of approximately 79% in this cohort, this high rate could be explained by failure to recover spontaneous cardiac activity during extracorporeal warming in 26 patients. The authors showed an increased in mortality in the group of patients rewarmed by the cardiopulmonary shunt without VA-ECMO. Thus, in the 33 patients with ROSC, the survival rate with a good neurological outcome was approximately 56% in patients with VA-ECMO, compared to 18% in patients with extracorporeal circulation (ECC). Moreover, the authors reported 9 deaths from massive pulmonary oedema in the ECC group, while there were no deaths in the VA-ECMO group. This study shows an increased mortality rate in the ECC group supported by the multivariate analysis. Indeed, VA-ECMO support was demonstrated as an independent risk factor of survival with an odds ratio (OR) of 6,6. The mechanisms underlying this increase in mortality rate can be explained by the occurrence of severe diastolic dysfunction of the left ventricle during rewarming and by ischemia-reperfusion injury. VA-ECMO, by giving the opportunity to provide prolonged hemodynamic support, could prevent this acute failure [9]. The results of this study and the others discussed below are presented in Table 2.

**Table 2:** Studies presented in discussion who compared results of ECLS

Studies	Type of study	Number of Patients	ECLS	Survival with good neurological outcome (%)
Althaus et al. [8]	Cases report	3	3 CPB	3 (100)
Ruttman et al. [9]	Retrospective cohort	59	25 VA ECMO 34 CPB	9 (36) 3 (9)
Kosiński et al. [10]	Retrospective cohort	31	31 VA ECMO	16 (52)
Mair et al. [11]	Retrospective cohort	22	22 CPB	2 (9)
Pasquier et al. [12]	Review of literature	286	85 VA ECMO 201 CPB	36 (42) 70 (35)
Saczkowski et al. [13]	Review of literature	658	290 VA ECMO 368 CPB	144 (50) 121 (33)

*ECLS* Extracorporeal life support, *CPB* cardiopulmonary bypass, *VA-ECMO* veno-arterial extracorporeal membrane oxygenation

This post-rewarming heart failure was found in various studies with different results. In experimental studies on canine or pig models, residual left systolic ventricular dysfunction was demonstrated [14, 15]. However, in a more recent prospective work conducted on a small sample of 9 patients in which VA-ECMO was implemented for HT III or IV, the researchers discovered moderate biventricular diastolic dysfunction but no systolic impairment after the rewarming process [16].

In our case-report, there was no reported cardiac systolic or diastolic dysfunction in the control echocardiography performed on day 2 after the rewarming process. The cardiac sequelae do not seem to be systematic. The mechanisms and the various forms of injuries still remain to be confirmed by a study on larger sample group.

There are no defined international guidelines concerning rewarming rate after VA-ECMO implementation. More studies on the neurological clinical evolution of patients who underwent a rewarming process must be carried out, in particular, with regard to the rate of the rewarming process. Only two experimental studies have reported central neurological sequelae in the event of abrupt rewarming in profound hypothermia. These studies used extremely rapid rewarming models, such as 12 °C in 4 min in the work of Enomoto et al., which was conducted on rabbits, and 20 °C in 20 min in a murine model in a study conducted by Gordan et al. [17, 18].

ECLS for HT IV patient is able to substantially improve survival rate by 21 to 100% according to the literature. Above all, there is a trend of an improved survival rate when VA-ECMO is the implemented ECLS mechanism [9, 19]. In a recent literature review on ECLS and HT IV patients, researchers found a global survival rate of 73%, with 89% of these patients showing a promising neurological recovery i.e., Cerebral Performance Category (CPC) 1 or 2 [20]. In this group, the researchers decided to only consider bystander-witnessed cardiac arrest patients, especially during the initial mobilization. This made it possible to eliminate patients already in cardiac arrest at the initial management, and for whom it is difficult to know whether the cardiac arrest is induced by the profound hypothermia or due to a hypoxic cause, which makes the global prognosis poorer. Despite the retrospectivity bias, this analysis demonstrates the good outcome of early management of profound hypothermia by ECLS, even in the case of early-stage cardiac arrest.

These high survival rates with good neurological outcomes, even in the case of prolonged resuscitation, are thanks to the massive decrease in cerebral metabolism during deep hypothermia. The cerebral metabolic rate of oxygen is reduced by 6% for each 1 °C decrease in core body temperature. This allows the brain cells to tolerate circulatory arrests 10-times longer than usual [21].

In a retrospective trial involving 31 patients, the delay of cardio-pulmonary resuscitation before implementing VA-ECMO ranged from 107 to 345 min, with 16 survivors, all of whom showed good neurological outcome (CPC1 or 2) [10]. For our patient, the time needed to implement VA-ECMO was approximately 260 min from the initial management and the start of the supportive circulatory treatment (low-flow time with CPR and external heart massage was 45 min), which did not prevent a full neurological recovery.

However, is it truly necessary to implement ECLS with every patient with severe hypothermia? The post-hoc analysis of resuscitation failures and deaths among these patients identified a number of independent risk factors.

Of these risk factors, kalaemia seems to be the most relevant. Considerable hyperkalaemia is related to a worse outcome due to a supposed underlying prolonged hypoxia/anoxia, which results in ischemia and cell lysis. International guidelines recommend proceeding to resuscitation and implementing VA-ECMO when there is a serum potassium level lower than 12 mmol/L, with an exception for avalanche hypothermia, where the cut-off value is 8 mmol/L. In this specific case, there is a higher risk of asphyxia and hypoxia before circulatory arrest, which jeopardizes the prognosis [1, 6]. These cut-off values are primarily based on published cases with good survival rate and neurological outcomes [22, 23].

Other parameters have been selected by the European Resuscitation Council to evaluate the futility of resuscitation in the case of HT III or HT IV, e.g., the concomitant presence of severe head injury, cerebral haemorrhage and/or significant comorbidities [6]. Some studies suggest that the level of initial lactate or pH as a poor prognosis factor, but these parameters were not selected by the group of experts because of the lack of evidence in other published cohorts [11, 24]. Favourable and adverse prognosis factors are presented in Table 3.

**Table 3:** Favorable and adverse prognostic factors in the literature for deep hypothermia resuscitation

Favorable factor	Adverse factor
Kalemia < 8 mmol per liter No cardiac arrest associated Female	Kalemia > 12 mmol per liter Asystole Male Severe head injury concomitant High comorbidity Cerebral hemorrhage associated Initial pH Initial lactate

More recently, two distinct medical teams tried to identify and regroup prognostic factors for good neurological outcome in predictive survival scores. Based on a retrospective analysis of 286 patients in 18 publications, Pasquier et al. developed the HOPE score, which takes into account six clinico-biological variables who are age, gender, asphyxia associated, CPR duration, serum potassium level and temperature (score calculation and predicted survival use an algorithm which is available online at: www.hypothermiascore.org). The statistical analysis showed that the area under the ROC curve for the HOPE score is more useful than kalaemia for predicting survival [12]. Saczkowski et al. published an another review screening 658 patients from 40 clinical cases and 44 retrospective cohorts. The survival rate was approximately 46%, with a good neurological outcome rate of 87,5% among the survivors (CPC 1 or 2). Three independent risk factors were identified in these cohorts: the initial hyperkalaemia and previous asphyxia before cardiac arrest were related to poor prognosis, whereas female sex was related to better prognosis. On the basis of these three variables, they defined the ICE score to determinate a survival with good neurological prognosis, which ranges from 85 to 0% in this model. Correlated to an area under the ROC curve of 0,849 [13]. Components of these two scores are presented in Table 4. Limiting factors for HOPE and ICE scores exist, as there has not yet been external validation, and potential publication bias is possible.

**Table 4:** Components of the HOPE Score and ICE Score

HOPE Score [12]	ICE Score [13]
Gender Asphyxiation with hypothermia First serum potassium Age Cardiopulmonary resuscitation duration Core temperature at admission	Gender Asphyxiation with hypothermia First serum potassium

In our case, the initial serum potassium level was 4 mmol/L, which is significantly below the 12 mmol/L cut-off. However, the lactate level was high, and the pH was below 7, which did not prevent good clinical and neurological outcomes. HOPE and ICE scores were not available when we managed our patient but if we calculated them, the HOPE score resulted in 59% survival, and the ICE score was 0 (0 point for male sex, 0 point for no associated asphyxia and 0 point for the initial serum potassium concentration less than 5 mmol/l), corresponding to a probability of survival with a good neurological outcome of 60% [12, 13]. These results, similar for the two predictive scores, raise the question of their usefulness in clinical practice. Indeed, is there any existing survival score that can help us predict when to proceed to a prolonged resuscitation and implement VA-ECMO? The dilemma is then to counterbalance the predicted low survival rate against the cost of VA-ECMO implementation, and therefore, assess life-limit on a strictly economic basis. On the other hand, is there still an indication to implement such an expensive technique even if predictive scores show low survival rates under 60 or 70%? These questions raise moral and economic questions and will require a reflection process, but first we need studies to approve these survival scores, and also to confirm the place of VA-ECMO in deep hypothermia. The development of new ECMO circuits that are easy to install and start will also be important in the years to come in order to make this technique more accessible and less expensive. Nevertheless, patients, after that ECMO is implanted, should be managed by an ICU expert team of a referral hospital [25].

## Conclusion

Accidental severe hypothermia is a pathology with a high risk of cardiovascular collapse. However, with appropriate initial resuscitation, patients present good neurological outcomes. The studies reveal that VA-ECMO circulatory support seems to be the benchmark treatment to take over central rewarming in the international recommendations. Medical teams need to become aware of this less well-known treatment. However, scientifically speaking, we still need to develop some tools to identify patients eligible for this technique and balance the economic and medical limitations.

## Data Availability

The datasets used and/or analysed during the current study are available from the corresponding author on reasonable request.

## Abbreviations

CPB: Cardiopulmonary Bypass
CPC: Cerebral Performance Category
CPR: Cardiopulmonary Resuscitation
ECC: Extracorporeal Circulation
ECLS: Extracorporeal Life Support
ECMO: Extracorporeal Membrane Oxygenation
HT III: Hypothermia degree III
HT IV: Hypothermia degree IV
LVEF: Left Ventricular Ejection Fraction
OR: Odds Ratio
ROSC: Return of Spontaneous Circulation
VA-ECMO: Veno-Arterial Extracorporeal Membrane Oxygenation
VTI: Velocity Time Integral
